# Neuronal Recordings with Solid-Conductor Intracellular Nanoelectrodes (SCINEs)

**DOI:** 10.1371/journal.pone.0043194

**Published:** 2012-08-15

**Authors:** Matthew R. Angle, Andreas T. Schaefer

**Affiliations:** Behavioural Neurophysiology, Max Planck Institute for Medical Research, Heidelberg, Germany; Baylor College of Medicine, United States of America

## Abstract

Direct electrical recording of the neuronal transmembrane potential has been crucial to our understanding of the biophysical mechanisms subserving neuronal computation. Existing intracellular recording techniques, however, limit the accuracy and duration of such measurements by changing intracellular biochemistry and/or by damaging the plasma membrane. Here we demonstrate that nanoengineered electrodes can be used to record neuronal transmembrane potentials in brain tissue without causing these physiological perturbations. Using focused ion beam milling, we have fabricated Solid-Conductor Intracellular NanoElectrodes (SCINEs), from conventional tungsten microelectrodes. SCINEs have tips that are <300 nm in diameter for several micrometers, but can be easily handled and can be inserted into brain tissue. Performing simultaneous whole-cell patch recordings, we show that SCINEs can record action potentials (APs) as well as slower, subthreshold neuronal potentials without altering cellular properties. These results show a key role for nanotechnology in the development of new electrical recording techniques in neuroscience.

## Introduction

Intracellular recording is essential for investigating the electrical activity that occurs below a neuron’s threshold for AP generation, and so it is an important tool for elucidating the mechanisms by which synaptic inputs are transformed into AP output [1]–[4]. Intracellular techniques have led to critical insights into the biophysics of dendritic integration [5] and action potential generation [6], but measurements of the subthreshold activity underlying perception, memory and behavior have been limited due to the difficulty of establishing minimally invasive, stable intracellular recordings *in vivo* [7].

Intracellular electrodes often cause biochemical and physiological perturbations in the cells from which they are recording. In whole-cell patch recording [8], [9], for example, the patch pipette internal solution rapidly exchanges solutes with the cytoplasm. This allows for the experimental control of intracellular concentrations of ions and other molecules by using different pipette internal solutions [10], but it also leads to the disruption of biochemical processes that are necessary for normal cellular function [11]–[17]. Dilution of freely diffusible cellular molecules occurs within a few minutes, making dialysis a limiting factor for reliable whole-cell recording [18], [19]. Using a variation of the whole-cell patch clamp technique, perforated patch recording [20], [21], one can avoid the unwanted dialysis of biomolecules, but poor sealing, changing access resistance and spontaneous membrane ruptures have limited the *in vivo* application of this technique (but see [22]).

Another type of intracellular recording electrode, the sharp microelectrode, differs from a patch pipette in that it enters the cell by piercing through the plasma membrane [23]–[26]. Sharp electrodes have smaller tips than patch pipettes and therefore dialyze the cytoplasm more slowly, but the impalement often results in irreparable membrane damage and shunting of the membrane potential [27]–[32]. In a study where whole-cell recordings were established prior to sharp microelectrodes penetration, all neurons showed significant depolarization following impalement [32].

One way to avoid the dialysis of cellular molecules by electrode internal solution would be to replace the liquid, electrolytic conductor in the micropipette with a solid, metallic conductor [33]. Incidentally, metallic microelectrodes aren’t new to intracellular recording [34], and their use actually precedes the first use of electrolyte-filled microelectrodes [23], [24]. The first *in vivo* neuronal recordings using metal microelectrodes were probably the putative intracellular recordings that were reported by Hubel in 1957 [35], which occurred occasionally while recording extracellular units with tungsten microelectrodes. Unfortunately, though solid-conductor intracellular microelectrodes would be non-dialyzing, they would be just as likely to introduce a leak current upon impalement as electrolyte-filled pipettes, thus mitigating their advantage over patch pipettes with respect to dialysis. To make solid-conductor intracellular electrodes practical for long-term neuronal recording, they must first be engineered in such a way as not to cause a membrane shunt upon insertion.

Developments in nanotechnology have prompted scientists to consider new, nanoengineered tools to access the interior of cells for intracellular electrical recording [36]–[46]. The challenge to neurophysiologists has been to integrate these nanotechnologies into recording electrodes that are also resilient and maneuverable enough for use in brain tissue. This has proven difficult, largely because the wafer-based nanofabrication processes used to produce many nanostructured devices limit their use to flat preparations such as dissociated cell cultures. Only a few devices have been developed that would be suitable for being delivered into deep tissue (e.g. [38] and [46]). Additionally, there has been no direct demonstration that any of these tools can be used to record from neurons without disrupting their physiology, which requires that properties such as resting membrane potential and input resistance be independently measured before and after membrane penetration by the nanoelectrode. Because reducing invasiveness is a key motivation behind developing new nanotechnology-based tools for electrophysiology, such tests will be critical in evaluating first-generation nanoelectrode technologies.

Here we describe Solid-Conductor Intracellular NanoElectrodes, or SCINES. We report their fabrication, electrical characterization, and use for recording neuronal transmembrane potentials in brain slices. We also perform simultaneous whole-cell recordings to independently assess the invasiveness of SCINE recording.

## Results

**Figure 1:**
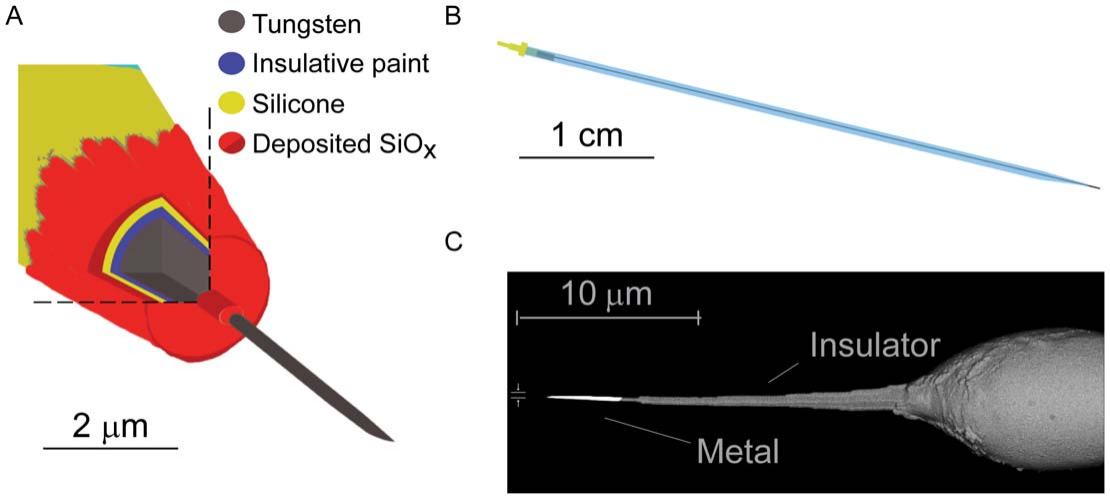
SCINE and manipulator design. **A**. Magnified cross-sectional drawing of the SCINE tip. **B** SCINE overview showing the pulled glass capillary and the gold pin for the electrical connection to the amplifier. **C** Scanning electron microscopy image using the backscattered electron detector for material contrast (metal vs. insulator). Width dimension  =  300 nm.

We constructed tungsten SCINEs by milling conventional tungsten microelectrodes [35] using a focused ion beam to 100–400 nm in diameter for a length of several microns. To ensure that electrical contact was restricted to the nanoscopic tip, SCINEs were then reinsulated at the base of their tips using electron-beam-assisted chemical vapor deposition (Figure 1A–C, Figure S1). Additionally, they were chemically modified using a hydrophobic silane reagent in order to facilitate their penetration into cells [47] (see Methods for details).

**Figure 2:**
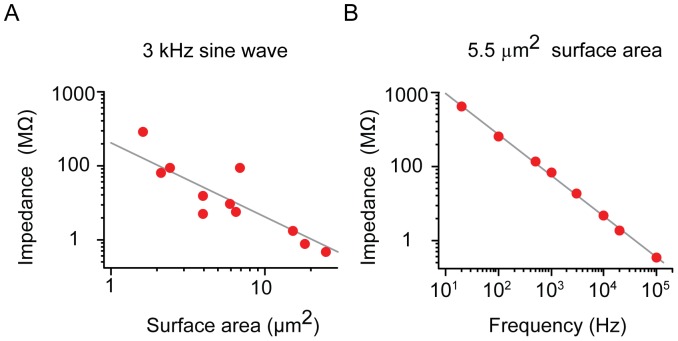
SCINE impedance. **A**. Impedance as function of exposed surface area, measured at 3 kHz; line indicates fit Z ∝ 1/A. **B** Electrode impedance as a function of frequency. For this example, the exposed tungsten surface area was 5.5 µm^2^. Line indicates fit Z ∝ f^−(1−α)^ with α = 0.45.

We measured the electrical properties of tungsten SCINES, namely the electrochemical impedance of microscopic tungsten surfaces in aqueous electrolyte (pipette internal solution). This impedance is inversely related to surface area (Figure 2A) and decreases as a function of frequency Z ∝ f^− (1−α)^ with α = 0.30±0.16 (n = 7, Figure 2B); DC resistances were greater than 100 GΩ. Similar properties of metal–electrolyte interfaces have been described previously for larger surfaces [48], [49]. The high impedance of small-surface-area SCINES forms a voltage divider with all of the stray impedances in the recording setup. To maximize signal amplitude, we have therefore systematically reduced all sources of stray capacitance: Electrode capacitance has been reduced to <0.3 pF by a careful insulation procedure and all SCINE recordings were performed using a custom-built, low noise, low input capacitance amplifier (see Methods), resulting in a total stray capacitance of <4 pF.

**Figure 3:**
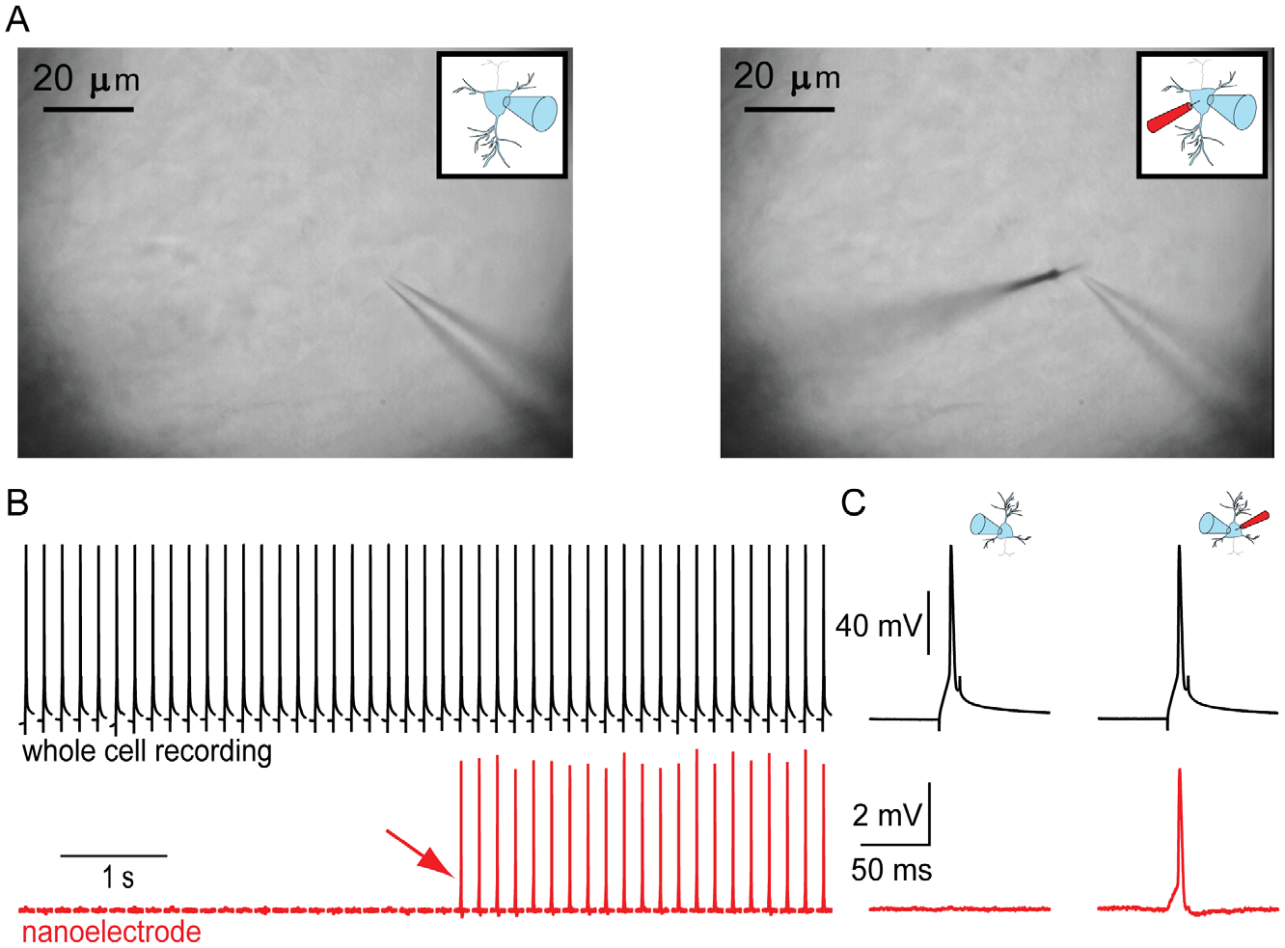
SCINE recording. **A**. (left) Differential Interference Contrast (DIC) Micrograph of a patch-clamped neuron with the nanoelectrode above the focal plane. (right) Double recording from the same neuron after the SCINE is inserted into the patch-clamped neuron. **B** Simultaneous whole-cell patch (black) and SCINE (red) recording from a pyramidal neuron in a rat hippocampal slice culture. The red arrow indicates when the SCINE penetrated the neuronal membrane. Action potentials (APs) were evoked via the whole-cell electrode. Gaps between traces are approximately 100 msec. **C** Comparison of evoked action potentials in whole-cell and SCINE channel before (left) and after (right) membrane penetration by the SCINE. All traces shown are single, unaveraged traces that are low-pass filtered at 5 kHz. SCINE recordings are corrected for baseline drift.

**Figure 4:**
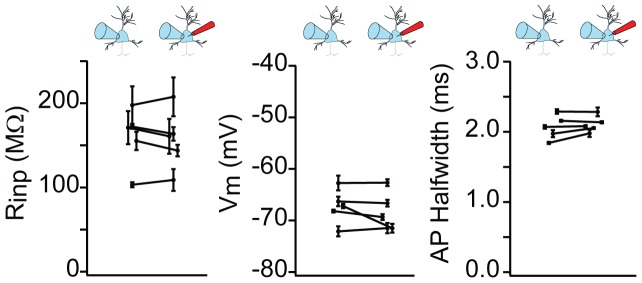
SCINEs can be inserted into cells non-invasively. Examples (n = 5) where SCINE recordings did not significantly alter cellular properties (measured from whole-cell electrode). Comparison of input resistance, membrane potential and AP half width as measured with the whole-cell electrode before and after SCINE membrane penetration (error bars are 1 standard deviation). Overall means were: R_inp_ = 160±12 MΩ (mean ± s.d., before), 157±14 MΩ (after), p = 0.53 (paired t-test); V_m_ =  −67.3±0.8 mV; 68.3±0.7 mV; p = 0.31; APHW  =  2.11±0.11 ms; 2.13±0.09 ms; p = 0.64.

To assess the ability of SCINES to record sub- and suprathreshold neuronal membrane potentials, we performed combined patch pipette and SCINE recordings in brain slices (Figure 3A–C). After obtaining a stable whole-cell recording, we inserted a SCINE into the same neuron under visual control (Figure 3A, right). Upon penetration, the SCINE signal increased dramatically (Figure 3B,C). The whole-cell recording provided a measurement of physiological properties, such as action potential half-width, resting membrane potential, and input resistance, which could be compared before and after the insertion of the SCINE. While SCINE penetration often resulted in the loss of cell input resistance and membrane potential (Figure S2), in ∼10% of attempts the silane-functionalized SCINE inserted through the plasma membrane without altering cellular physiological properties at all. For the five recordings shown in Figure 4, the mean change in input resistance was 3.0±10.0 MΩ (mean ± s.d.; −1.6±6.3%), the mean change in membrane potential was −1.0±2.0 mV (−1.5±2.9%), and the mean change in action potential half-width was 21±93 µs (1.4±4.5%).

Our experiments indicate that surface chemistry can critically improve SCINE penetration into neurons; only those SCINEs that were treated with hydrophobic silane were able to record intracellularly without causing a change in cellular properties. Unmodified SCINEs only rarely inserted into neurons and in these cases only after substantial indentation of the neuronal membrane. Subsequent penetration resulted in a change of input resistance and membrane potential (Figure S3), consistent with observations from sharp electrode recordings, that indentation prior to penetration often predicts membrane damage [25].

**Figure 5:**
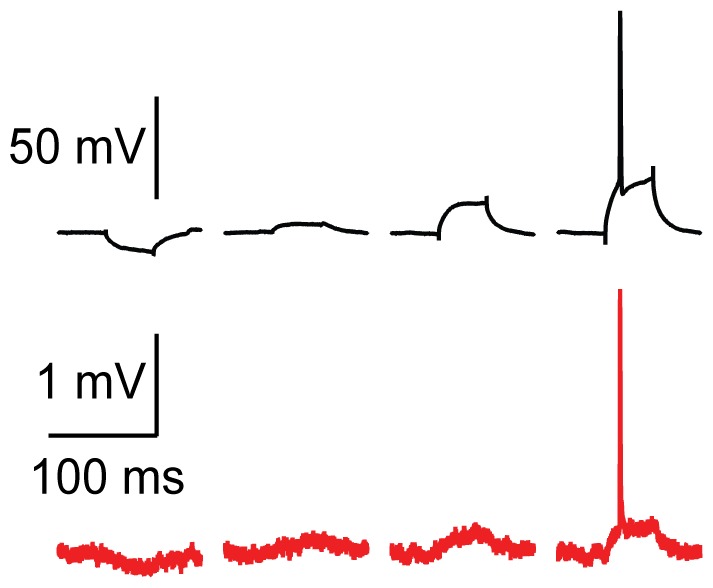
SCINEs can record both APs and slower, subthreshold potentials. SCINE recording (red) of subthreshold and suprathreshold signals evoked by a simultaneous whole-cell recording electrode (black). In the far right trace, the AP height and depolarizing step response are 109 mV and 27 mV above RMP, respectively. The SCINE recording measures 2.62 mV and 0.25 mV for the same features; the RMS of the noise in the first 100 ms of the SCINE recording (before current injection) is 0.05 mV. All traces shown are single, unaveraged traces that are low-pass filtered at 5 kHz. SCINE recordings are corrected for baseline drift.

Though SCINE signal amplitudes were attenuated due to the voltage divider formed by the high impedance metal-electrolyte interface and the stray capacitance in the recording setup, the electrode filtering did not seriously distort the action potential waveform (Figure S4) or prevent the measurement of successive action potentials (Figure S5). Futhermore, SCINEs were able to measure both action potentials and low-frequency subthreshold signals in single trials (Figure 5), due to the low noise in the recordings (rms 55±9 µV [mean ± s.e.m., n = 5], 20 Hz-25 kHz).

The SCINE recordings began with SCINE penetration and were considered “lost” when the spike amplitude dropped below the detection limit or when the SCINE recording reverted to an apparently extracellular recording, whichever occurred earlier (see Methods). For the recordings shown in Figure 4, the durations were between 26 and 101 seconds (47±31 sec, mean ± s.d., n = 5). After this signal loss, it was possible in some cases to re-penetrate the cell and recover the signal. Furthermore, in the case where SCINE penetration damaged the plasma membrane (e.g. Figure S2), electrical coupling did not diminish without a concomitant recovery of input resistance. These together suggest that the signal decay was due to membrane resealing around the SCINE, rather than due to electrode fouling by some adsorptive or electrochemical process. One possible mechanism for resealing around the SCINE could be membrane-spreading along the hydrophobic surface, similarly to what has been reported for silanized sharp microelectrodes [47]. Most importantly, although the SCINE recording was lost over a period of seconds to minutes, the whole-cell recording remained unaffected (Figure S6).

## Discussion

Here we have demonstrated that SCINEs can be inserted into neuronal membranes in brain tissue and can, in some instances, record changes in the transmembrane potential without measurably altering cellular properties. The solid construction prevents SCINEs from dialyzing neurons, thus eliminating the fundamental limit imposed on recordings by electrodes with aqueous electrolyte conductors. The fact that SCINEs are macroscopically identical to metal microelectrodes means that they are easily maneuverable and suitable for use in brain tissue, a feature not shared by many chip-based nanotechnologies. In fact, while nanoscopic metal tips are potentially sensitive to lateral movements, the hardness of the tungsten ensures that SCINEs can be repeatedly inserted millimeter-deep into brain tissue without noticeable damage to the tip, as long as the dura mater is removed and SCINE movements are strictly longitudinal. Current design employs glass as a sheathing material for the body of the SCINE. Exclusive use of e.g. silicone as an insulator could ensure that electrodes do not risk shattering in tissue, thus rendering SCINEs a potentially safer technique for *in vivo* use than conventional glass electrodes.

While we show a proof-of-principle for SCINE recording, several technical challenges must be solved before SCINEs become practical for routine neurophysiological investigation. The quality of SCINE intracellular recordings decayed significantly over periods of seconds to minutes. This, as well as their variable insertion success, indicates that further physicochemical stabilization of the SCINE-membrane interface is needed. Rational optimization of the existing interface, however, would be difficult due to the fact that the exact arrangement of the silane molecules on the SCINE surface is unknown, and consequently, the mechanism of interaction between the SCINE and lipid-bilayer is not well defined. In the future, a more controllable approach to surface modification may be necessary [41].

Another limitation of SCINEs is their high attenuation of neuronal signals, which would currently prevent the measurement of all but the largest synaptic inputs (Figure 5). This attenuation may be due to SCINEs incomplete insertion of their exposed tips through the plasma membrane. Based on the surface areas of the SCINEs used in this study (2–10 µm^2^) and the system stray impedance (<4 pF), our impedance measurements would have predicted signals of roughly an order of magnitude larger amplitude. If signal attenuation is indeed higher due to incomplete membrane insertion, then signal strength could be improved by optimizing the electrode-membrane interaction to favor complete insertion of the exposed metal tip. Secondarily, signal amplitudes might be further improved by using a different material for the exposed SCINE tip–one that forms a lower impedance interface in solution than does tungsten (e.g. iridium oxide [50]). Developing a lower-impedance interface would enable the measurement of low frequency signals and might also allow for the estimation of resting membrane potentials upon break-in. Furthermore, such an interface would better facilitate current injection, which is currently not practical using tungsten SCINEs.

Existing intracellular recording techniques have fundamental limitations that prevent them from being applied to study many important processes *in vivo*, so developing an innocuous, non-dialyzing electrode is a key technical goal for extending the timsescales of intracellular neurophysiology to those of neurophysiological processes. The recordings reported here are short-lived, but they demonstrate an approach to intracellular recording that can, in principle, be much less invasive to cells than currently available methods. Importantly, the loss of SCINE recordings is due to instability of the membrane-SCINE interface rather than the deterioration of cellular properties, implying that stabilization of the interface could lead to long-term intracellular electrical recordings that are free of the fundamental limitations of existing recording methods. We hope that this prospect will encourage nanoengineers and neurophysiologists to cooperate on future SCINE-based approaches to neuronal recording.

## Materials and Methods

### Ethics Statement

All animal experiments were performed according to European and German guidelines for the welfare of experimental animals and reviewed and monitored by the animal welfare officer (Tierschutzbeauftragte) of the MPI for medical research and the Abteilung 3 - Landwirtschaft, Ländlicher Raum, Veterinär- und Lebensmittelwesen of the Regierungspräsidium Karlsruhe.

### Fabrication

A 12–25 µm diameter tungsten wire (Advent Research Materials) was threaded into a standard 1–1.2 mm O.D., 90 mm long glass capillary. With the aid of a syringe, approximately 100 µl of conductive silver epoxy (CW2400 Chemtronics) was drawn up into the end of the capillary, contacting the wire. Next, a gold connector-pin (MK05, Reissig Electronic Vertriebs GmbH) was inserted into the epoxy-filled end of the capillary. After curing the epoxy at 165°C for >30 min, the glass was pulled on a Sutter P-97 Puller (Sutter Instrument Co.), and the protruding tungsten wire was trimmed and electrolytically etched to a conical, sub-micrometer tip [51]. The etching was performed under microscopic observation, in 4 N KOH, with an applied bias of (+) 4 to 20 V versus a platinum or tungsten loop electrode. The etchant solution was continuously replenished using a tube connected to a syringe. After washing with distilled, de-ionized H_2_O, the tips were dipped into an electrophoretic paint solution [52] made from 1 part glacial acetic acid and 9 parts Clearclad HSR (LVH Coatings). The tungsten tip was held at a potential of −4 to −7 V versus a silver/silver chloride counter electrode for one minute to coat it with the electrophoretic paint. The paint was cured at 165°C for 20 minutes. Afterwards, the probes were dip-coated under microscopic observation into a 3 mm diameter wire loop (tungsten wire, diameter 250 µm) containing a viscous silicone glue, Elastosil E41 or E43 (Wacker silicones). After the silicone cured (20–30 min at room temperature), the insulated electrodes were milled to nanoscopic proportions using the FIB [52] under SEM control (Neon 40 EsB Crossbeam, Zeiss). To avoid charging, the electrode holder for the FIB/SEM was designed in a way that the back pin was connected to the microscope ground. The electrode was first cut on two opposite sides to form a paddle shape. Next, the electrode was rotated 90° around its long axis and the paddle was shaped into a post. This rotation and cutting was repeated until the diameter of the post was between 100 and 400 nm (Figure S1). This size was selected on the basis of earlier experiments, done with nanoscopic atomic force microscopy probes [53], [54]. Larger, initial cuts were made using the 1 nA or 500 pA FIB aperture; the final thinning of the tip was accomplished with a 20 pA aperture. Exposed tungsten around the base of the tip was reinsulated using electron-beam-assisted chemical vapor deposition of insulative silicon oxide (EBCVD) [55]. This was accomplished using the electron microscope’s gas injection system to flood the volume around the electrode with a silicon dioxide precursor gas, 2,4,6,8,10-Pentamethylcyclopentasiloxane [56] (purchased from Zeiss), which was decomposed under the energy of the scanning electron beam to deposit insulative silicone dioxide. EBCVD takes approximately 10–20 minutes of deposition time per side of the electrode. Deposition time is inversely related to scanning speed and beam accelerating voltage. Coating was considered complete 5–10 minutes after the backscattered electron signal of the tungsten was no longer detectable. Cross-sections of these coated surfaces reveal silicon oxide layer thicknesses of 50–200 nm. The coated portion of the cylindrical tip is deliberately coated less than the base (only to the point where the backscattered electron signal of the metal is lost) in order to keep its diameter as small as possible. The completed SCINE was removed from the Crossbeam system and treated with oxygen-plasma in a plasma cleaner (Harrick Plasma) for 20–30 minutes to expose inorganic hydroxyl groups for reaction with the hydrophobic silane reagent.

### Silane Functionalization

After oxygen plasma treatment probes were dipped for 1–2 minutes into a 1–3% solution of benzophenone-silane (4-(3′triethoxysily) propylamidobenzophenone) in 95% ethanol. They were then rinsed with ethanol or acetone, and dried in an oven at 110°C to complete condensation of the silane on the SCINE surface. Exposure of SCINEs to silane solution for more than 2 minutes often resulted in a large increase in impedance, indicating a thick polymerization layer. For this reason, exposure was limited to less than 2 minutes. Before using the silane solution to treat SCINEs, the activity of the solution was first confirmed by treating a glass microscope slide and qualitatively observing a change of water contact angle on the slide before and after treatment: on glass surfaces, and especially plasma cleaned glass surfaces, a droplet of water spreads very thinly across the glass. On a hydrophobic surface the droplet will form a bead [57]. Benzophenone silane was synthesized according to ref. [58], with minor modifications as suggested by its corresponding author: 15 grams of the acid chloride of benzophenoic acid (Sigma) was dissolved in 75 ml of dry tetrahydrofuran. This was added slowly, over a period of 20–30 minutes, to a cold solution of 13.2 grams aminopropyltriethoxy silane (Sigma) and 6.7 grams triethylamine (Sigma) in 75 ml dry tetrahydrofuran (THF). The reaction was then allowed to warm over a period of several hours. The reaction was performed under inert gas to prevent polymerization of the silane. Afterwards, the product was quickly vacuum-filtered through filter paper to remove polymerization products. The remaining THF was removed by use of a rotary evaporator (<40°C). The crude product was re-dissolved in anhydrous ethanol and re-filtered. When stored as a solid, the silanes were more likely to polymerize. In our hands, benzophenone silane underwent a color change over time when dissolved in toluene. Thus, the silane was stored in anhydrous ethanol, and always verified for silane activity before use by glass slide treatment and water contact angle observation, as described above.

### Impedance Measurements

Capacitance measurements of the electrode-electrolyte interface were performed using a GW Instek LCR-821 lock-in amplifier that can measure small capacitances reliably (<0.01 pF). The positive and negative inputs to the LCR-821 were attached to the gold pin of a SCINE and a tungsten loop electrode, respectively. The SCINE was then manipulated using a manual micromanipulator to dip it into and out of a droplet of internal solution (see Electrophysiology) contained in the tungsten loop electrode. The device was zeroed with the electrode just above the solution, and the measurement was made with the electrode immersed in the solution. For all measurements, the LCR output voltage was a 5 mV sine wave.

To measure the frequency dependence of the metal-electrolyte interface, we cut off the tip of several insulated electrodes using the focused ion beam, and measured the capacitance of electrodes ranging from 1–33 µm^2^ in surface area at frequencies between 1 kHz and 100 kHz. Resulting capacitance measurements were then converted into impedance values (Z = (2πfC)^−1^). Independent confirmation of the frequency-dependent impedance was performed on the same electrodes using the Axoclamp-2B (Molecular Devices, Inc.) by voltage clamping the electrode in a bath of internal solution and measuring the current that flowed when the command voltage was Gaussian white noise. Dividing the Fourier transform of the command voltage by the Fourier transform of the measured current gave us the impedance as a function of frequency (data not shown). To measure impedance as a function of exposed surface area, we cut off the tip of an insulated electrode with the focused ion beam and measured its surface area from an electron micrograph using ImageJ (NIH). Impedance was assessed using the LCR at 3 kHz as described above. After exposure and measurement, all electrodes were re-insulated using electron-beam-assisted chemical vapor deposition of silicon oxide. The electrodes were again immersed in electrolyte solution, and the remaining stray capacitance was measured. This stray capacitance, which was less than 0.3 pF for all electrodes, was subtracted from the first capacitance measurement, giving the true capacitance for the metal-electrolyte interface at each exposed surface.

### Electrophysiology

Whole-cell patch recordings were performed in rat hippocampal organotypic slice cultures [59], [60] between 7 and 12 days *in vitro* using an Axoclamp 2-B, visualized using Diferential Interference Contrast (DIC) optics on an Axioplan 2 microscope (Zeiss) with a custom built stage containing a solution chamber for slice physiology. Electrode internal solution was: 130 mM K-methansulfonate, 7 mM KCl, 10 mM Hepes, 2 mM ATP (sodium salt), 2 mM ATP (magnesium salt), 0.5 mM GTP, 0.05 mM EDTA, pH = 7.4, Osmolarity  =  283 mOsm/kg. External recording solution contained: 125 mM NaCl, 25 mM NaHCO3, 2.5 mM KCl, 1.25 mM NaH2PO4, 25 mM Glucose, 2 mM CaCl2, 1 mM MgCl2. All experiments were performed at room temperature. SCINEs were connected to a custom-built amplifier and digitizer. Briefly, it uses a low input capacitance buffer amplifier (AD549, Analog Devices) and a high resolution, low-noise 24-bit Analog-to-Digital Converter (ADS1271, Analog Devices). The amplifier was not designed to pass current and acted only as a voltage-follower. Data was acquired from the digital output of the amplifier/digitizer using a National Instruments PCI-6229 card, Labview 2010 SP1 (National Instruments), Igor Pro 6.0 (WaveMetrics), and the Igor acquisition/analysis package developed by Jason Rothman, Neuromatic 2.00 (www.neuromatic.thinkrandom.com). Patch pipette and SCINE were both lowered into the bath solution and positioned by micromanipulator control to tens of micrometers above the slice surface. A suitable pyramidal neuron was selected, a patch recording was established (access resistance 8–20 MΩ) and an I-V protocol performed. Then repetitive action potentials were evoked by current injection with the patch pipette while the SCINE approached the patch-recorded cell. Shortly after penetration, another I-V protocol was performed for before-after comparison.

### Analysis and Signal Processing

For the before-after comparison of cellular properties, in 2 cells for which input resistance before and after penetration was reported, input resistance was calculated (R = V/I) from the average voltage V and current I from the last 5 ms of a 100 ms hyperpolarizing step. In the remaining 3, where the SCINE signal was lost before a post-penetration I-V protocol could be obtained, the change in input resistance was estimated by calculating the ratio of the rise times of the voltage responses to 10 ms depolarizing current injections, before and after penetration. The rise time τ is determined by the cell input resistance R and capacitance C, τ = RC, so that when cell capacitance remains constant, the ratio of the rise times will be the ratio of the input resistances. This analysis was repeated for the recordings where after-penetration I-Vs existed, and the two approaches yielded indistinguishable results. The resting membrane potential was calculated by averaging 35–50 ms before the respective input resistance current injection protocol. AP half width was calculated from averaging >10 APs before and after break-in, and calculating the time difference between the two points in the action potential where the potential was equal to half the difference between the action potential threshold and action potential peak.

For calculating recording duration, two time points were obtained for each recording: (1) AP amplitude duration – the time point at which the SCINE-measured AP amplitude consistently (in two repeated measures) dropped below the detection level, set as three times the rms of the noise in the SCINE recording. We define the time of the first of the two repeated measures as the AP amplitude duration. (2) Intracellular duration – the time point at which the SCINE-measured AP half width (measured from baseline to peak, measurements from threshold to peak did not appreciably alter the results) consistently (in two repeated measures) dropped below the half-width of an extracellular recording. The latter was estimated from calculating the half width of the derivative of the whole-cell recording trace. We define the time of the first of the two repeated measures as the intracellular duration. For obtaining a conservative estimate of intracellular recording duration we report the *earlier* of the two duration measures (AP amplitude and intracellular duration).

SCINEs have a large DC resistance (>100 GΩ), resulting in low-frequency baseline fluctuations. Where noted this drift has been subtracted for displaying purposes. This was accomplished in the following way: for the period of the current injection and action potential after-hyperpolarization, the points before and after this period were linearly interpolated to produce an AP-subtracted voltage record, which was low-pass filtered. This baseline was then subtracted from the original recording.

All recordings were sampled at 50 kHz and digitally low-pass filtered at 5 kHz.

## Supporting Information

Figure S1 **SCINE fabrication scheme.** This schematic shows the process of making SCINEs, as described in the Methods section.
(TIF)

Figure S2 **Unsuccessful SCINE penetration.** SCINEs do not yet work as reliably as sharp microelectrodes or patch pipettes; non-destructive membrane penetration occurs only in a minority of cases. **A** This double-recording typifies an unsuccessful attempt at SCINE recording (red). The whole-cell recording (black) shows that the cell input resistance drops and the membrane potential becomes depolarized when the SCINE pierces through the plasma membrane. **B** Expanded view of the gray-shaded period in A, also showing the current injection from the whole-cell pipette (middle). Two action potentials (arrows) are measured by the SCINE recording (red) before the neuron dies. All traces shown are single, unaveraged traces, low-pass filtered at 5 kHz.
(TIF)

Figure S3 **Untreated tungsten nanoelectrode. A** Untreated SCINES considerably deform the neuronal plasma membrane prior to penetration. **B** Recording from an untreated SCINE (red). The recording begins after the SCINE has already been pushed deeply into the neuronal cell body as schematized in A. The neuron is firing action potentials, which are evoked by somatic current injection by the patch pipette. After SCINE penetration, the whole-cell recording (black) shows that the membrane potential is depolarized by 16 mV due to a leak caused by the SCINE. Prior to penetration (first arrows), the action potential is high-pass filtered by the plasma membrane; after penetration (second arrows), the SCINE records an intracellular action potential waveform. **C** Comparison of successively evoked action potentials in whole-cell (black) and SCINE (red) channel just before (left) and just after (right) membrane penetration by the SCINE. All traces shown are single, unaveraged traces that are low-pass filtered at 5 kHz. SCINE recordings are corrected for baseline drift.
(TIF)

Figure S4 **Spike waveforms (single trials) from SCINE and whole-cell recording.** SCINE (red) and whole-cell recording (black) measurements of the same action potential are overlaid with each other to show the relative filtering properties of the two electrodes. Spikes are displayed for each of the cells shown in Figure 4. Traces are single, unaveraged traces.
(TIF)

Figure S5 **SCINE recording of multiple APs.** SCINE (red) and whole-cell recording (black) measurements of current-evoked action potentials. Traces are single, unaveraged traces.
(TIF)

Figure S6 **SCINE signal loss. A** This brief SCINE recording (red) and whole-cell recording (black) are annotated to highlight three time periods before membrane penetration (1), immediately after membrane penetration (2), and when the SCINE signal is diminishing (3). Throughout the entire duration, the whole-cell recording appears undisturbed (black). The time between traces is 130 ms. **B** The average of 10 consecutive whole-cell recordings of action potentials from the three time periods shown in Figure S6A. Note the spike shape remains constant between periods.
(TIF)
