# Supplementary figures and images for: Neuronal Recordings with Solid-Conductor Intracellular Nanoelectrodes (SCINEs)
Source: PLoS One. 2012 Aug 15;7(8):e43194. doi: 10.1371/journal.pone.0043194 (PMC3419643; doi:10.1371/journal.pone.0043194)

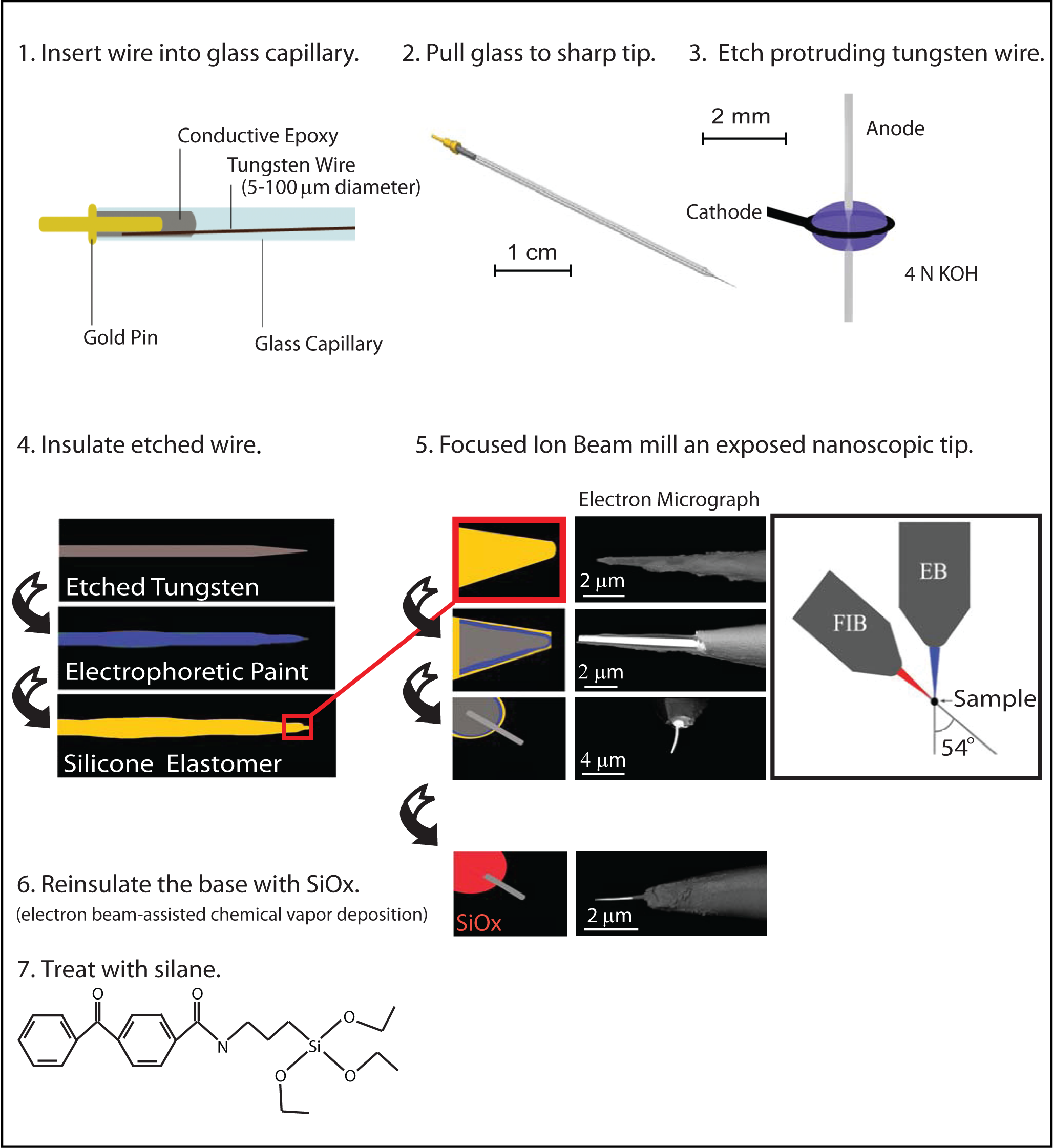

Supplement: Figure S1 — SCINE fabrication scheme. This schematic shows the process of making SCINEs, as described in the Methods section. (TIF) [file pone.0043194.s001.tif]

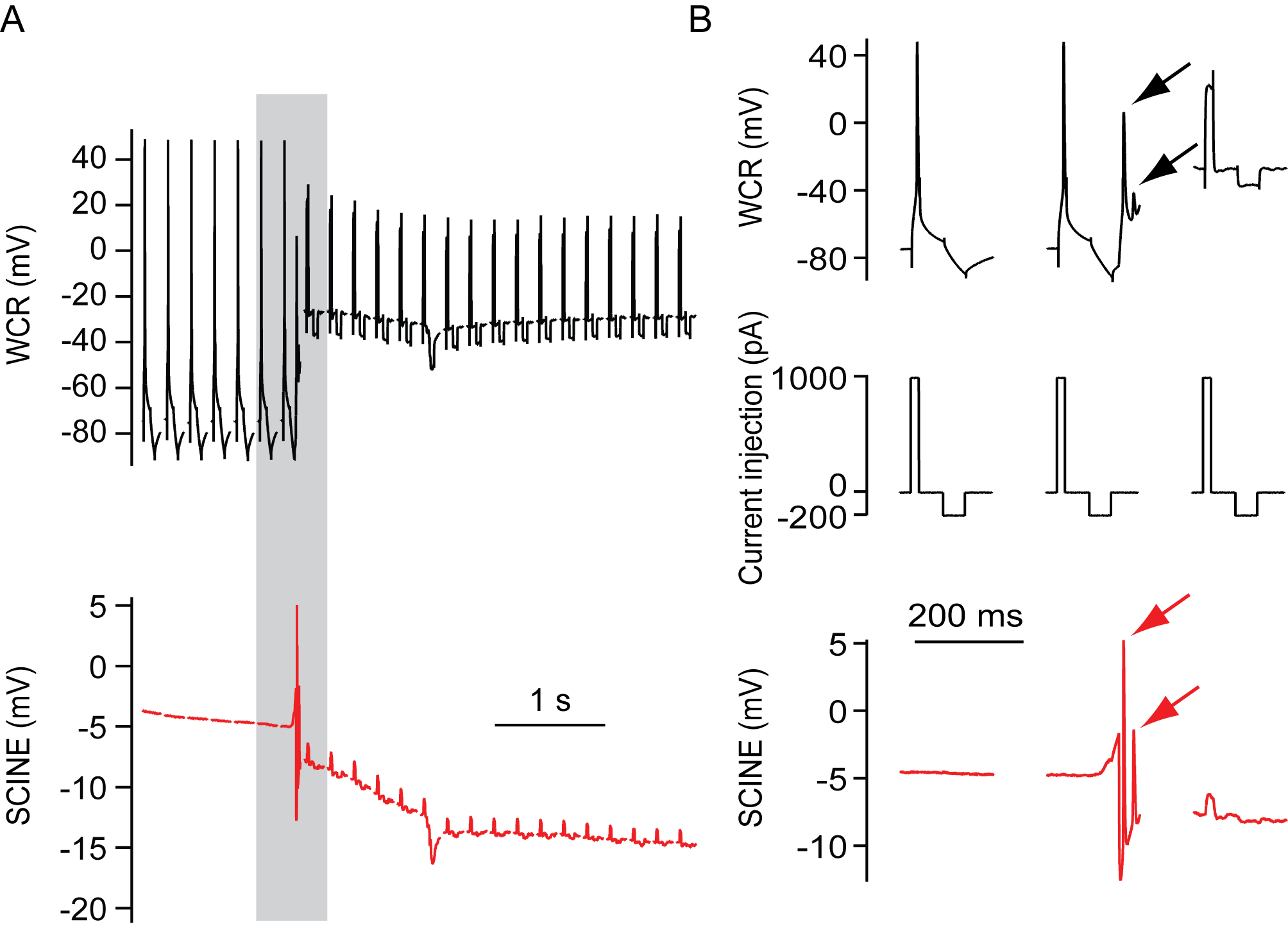

Supplement: Figure S2 — Unsuccessful SCINE penetration. SCINEs do not yet work as reliably as sharp microelectrodes or patch pipettes; non-destructive membrane penetration occurs only in a minority of cases. A This double-recording typifies an unsuccessful attempt at SCINE recording (red). The whole-cell recording (black) shows that the cell input resistance drops and the membrane potential becomes depolarized when the SCINE pierces through the plasma membrane. B Expanded view of the gray-shaded period in A, also showing the current injection from the whole-cell pipette (middle). Two action potentials (arrows) are measured by the SCINE recording (red) before the neuron dies. All traces shown are single, unaveraged traces, low-pass filtered at 5 kHz. (TIF) [file pone.0043194.s002.tif]

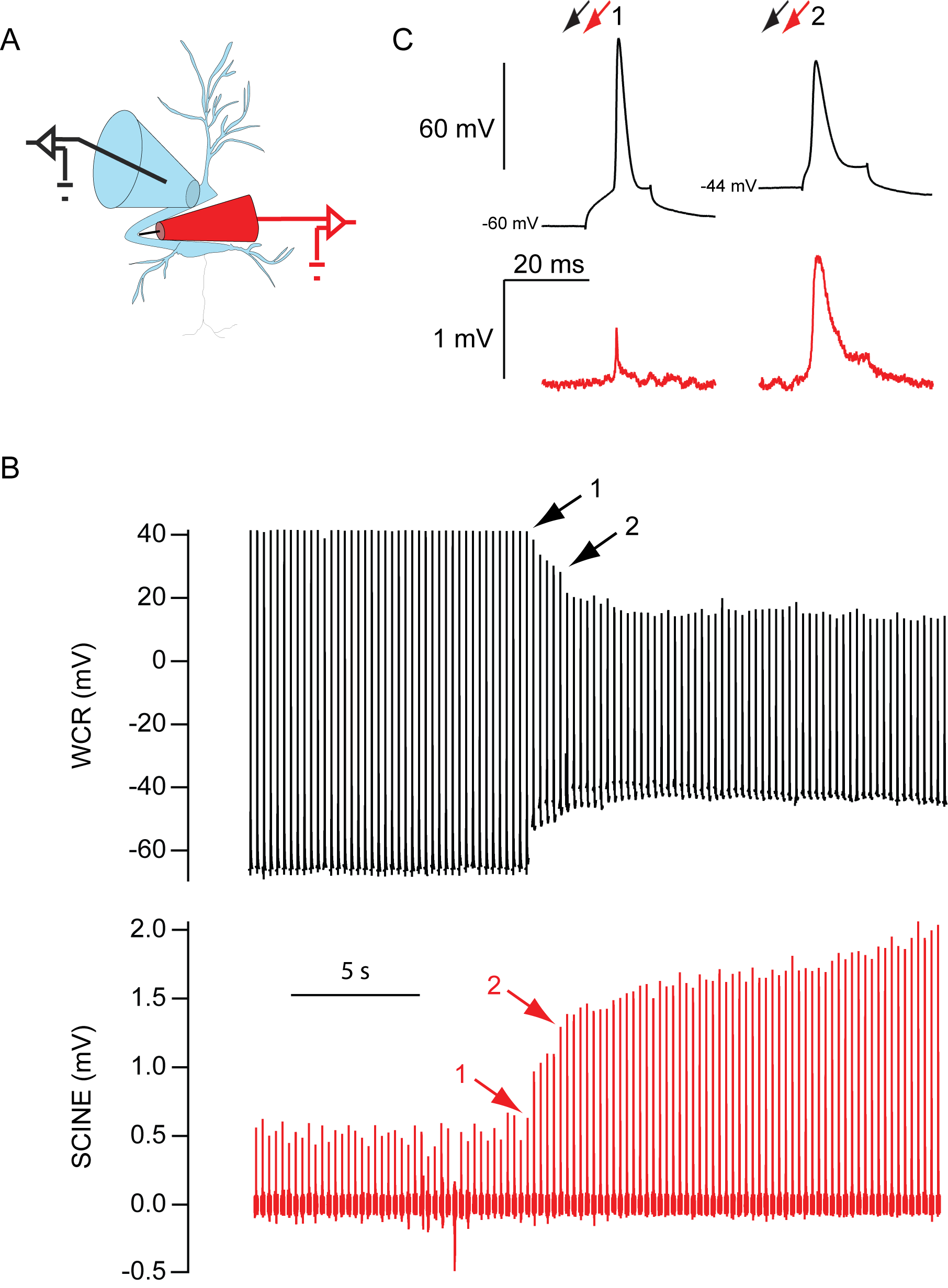

Supplement: Figure S3 — Untreated tungsten nanoelectrode. A Untreated SCINES considerably deform the neuronal plasma membrane prior to penetration. B Recording from an untreated SCINE (red). The recording begins after the SCINE has already been pushed deeply into the neuronal cell body as schematized in A. The neuron is firing action potentials, which are evoked by somatic current injection by the patch pipette. After SCINE penetration, the whole-cell recording (black) shows that the membrane potential is depolarized by 16 mV due to a leak caused by the SCINE. Prior to penetration (first arrows), the action potential is high-pass filtered by the plasma membrane; after penetration (second arrows), the SCINE records an intracellular action potential waveform. C Comparison of successively evoked action potentials in whole-cell (black) and SCINE (red) channel just before (left) and just after (right) membrane penetration by the SCINE. All traces shown are single, unaveraged traces that are low-pass filtered at 5 kHz. SCINE recordings are corrected for baseline drift. (TIF) [file pone.0043194.s003.tif]

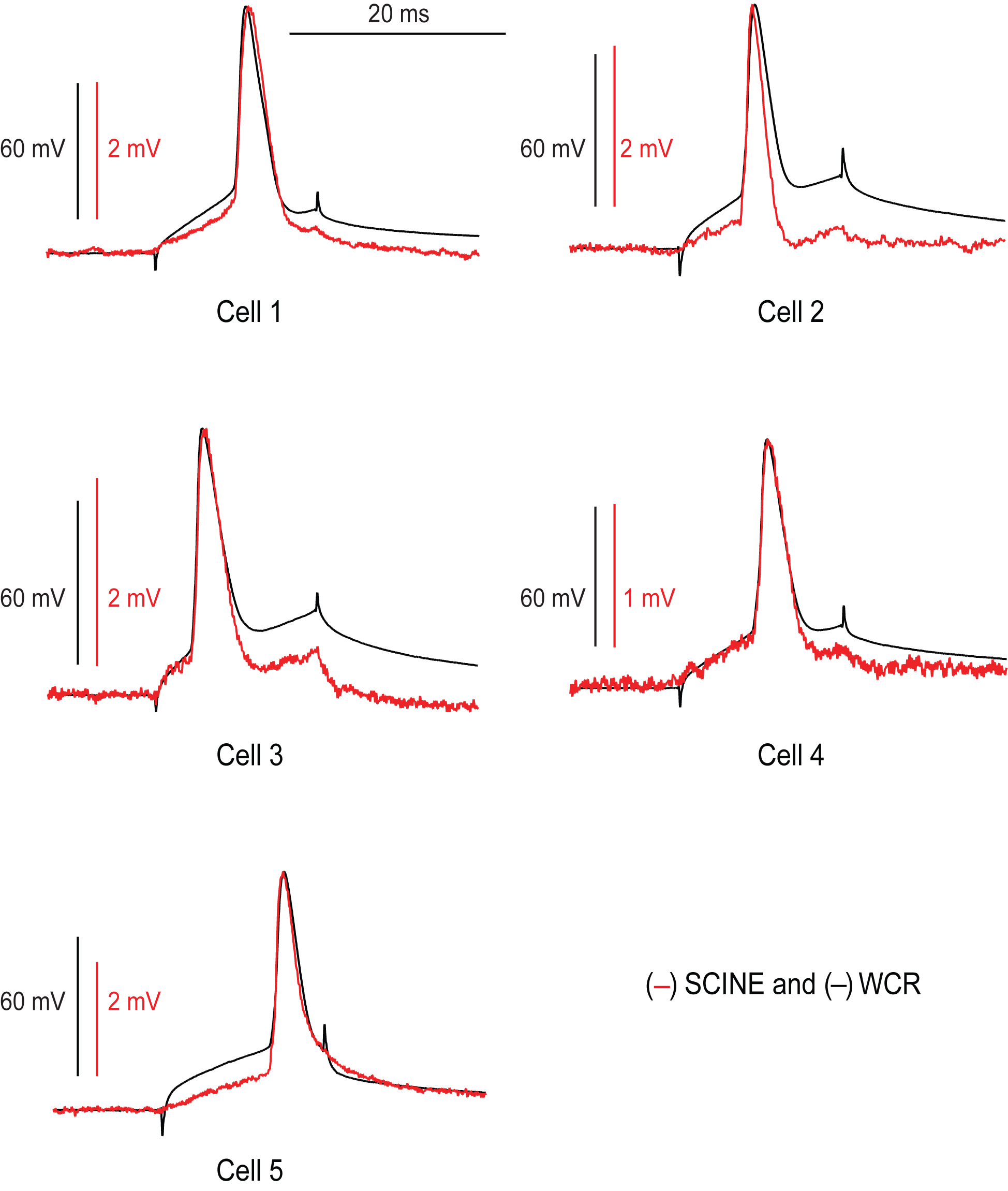

Supplement: Figure S4 — Spike waveforms (single trials) from SCINE and whole-cell recording. SCINE (red) and whole-cell recording (black) measurements of the same action potential are overlaid with each other to show the relative filtering properties of the two electrodes. Spikes are displayed for each of the cells shown in Figure 4. Traces are single, unaveraged traces. (TIF) [file pone.0043194.s004.tif]

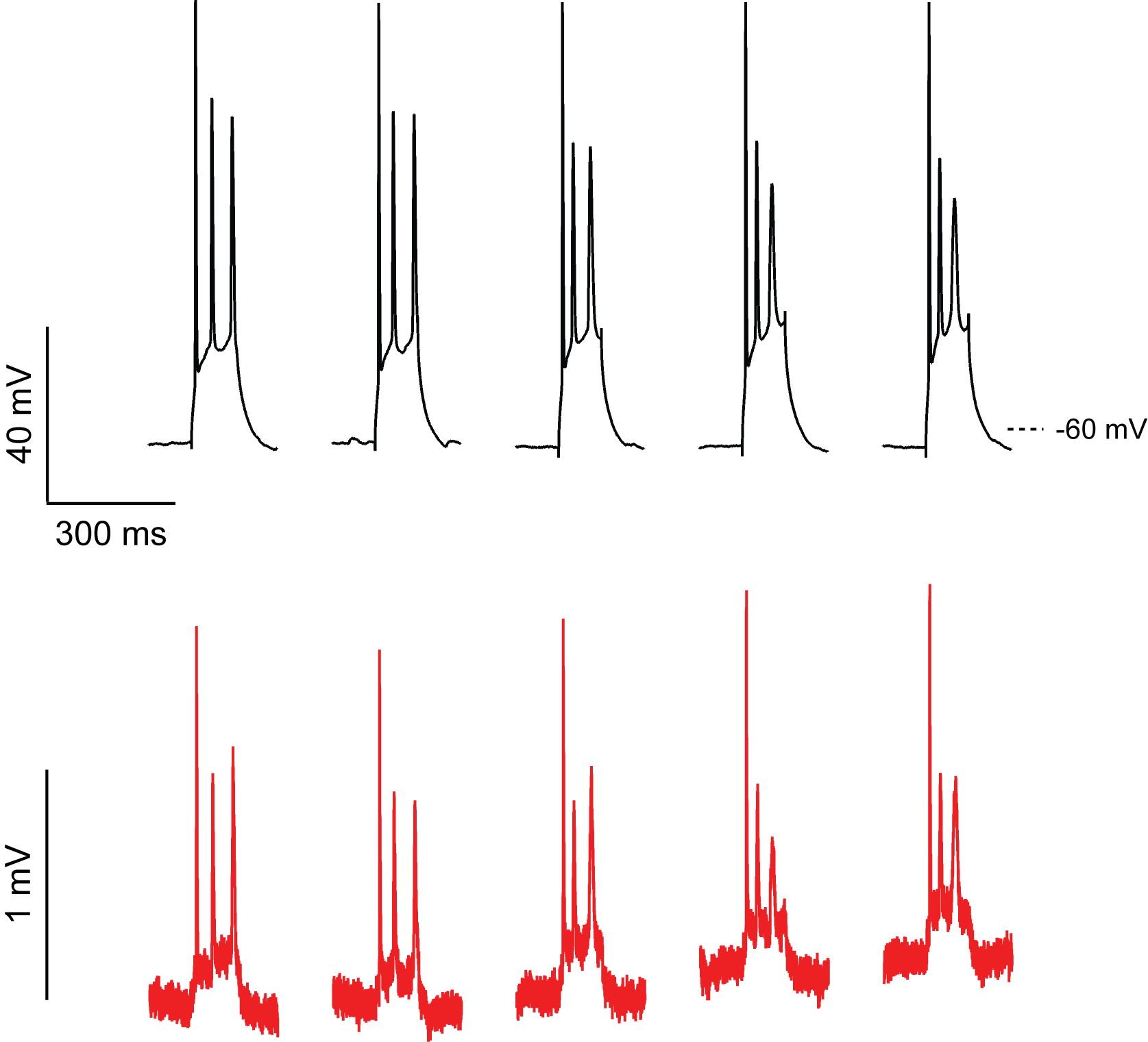

Supplement: Figure S5 — SCINE recording of multiple APs. SCINE (red) and whole-cell recording (black) measurements of current-evoked action potentials. Traces are single, unaveraged traces. (TIF) [file pone.0043194.s005.tif]

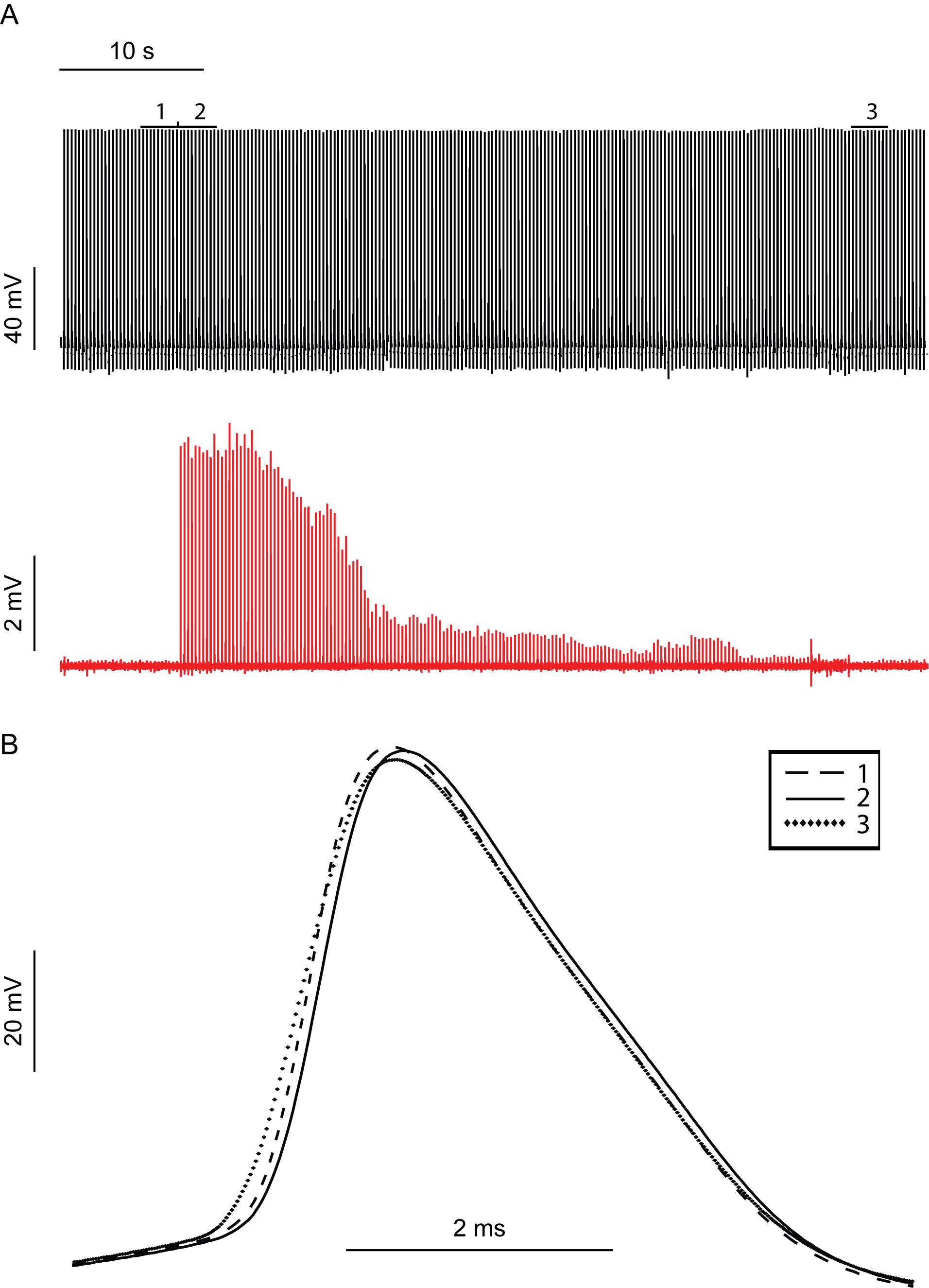

Supplement: Figure S6 — SCINE signal loss. A This brief SCINE recording (red) and whole-cell recording (black) are annotated to highlight three time periods before membrane penetration (1), immediately after membrane penetration (2), and when the SCINE signal is diminishing (3). Throughout the entire duration, the whole-cell recording appears undisturbed (black). The time between traces is 130 ms. B The average of 10 consecutive whole-cell recordings of action potentials from the three time periods shown in Figure S6A. Note the spike shape remains constant between periods. (TIF) [file pone.0043194.s006.tif]
